# Supplementary material for: Effectiveness of Therapeutic Patient Education Interventions in Obesity and Diabetes: A Systematic Review and Meta-Analysis of Randomized Controlled Trials
Source: Nutrients. 2022 Sep 15;14(18):3807. doi: 10.3390/nu14183807 (PMC9503927; doi:10.3390/nu14183807)
Supplement: Supplementary file 1 [file nutrients-14-03807-s001.zip › nutrients-1825079-supplementary.pdf]

Supplementary Table S1: Search strategy adapted for PubMed database

Concept	Keywords
**Intervention**	(“Health education” [MeSH] OR “patient education”[MeSH] OR “Health education” [ti/ab] OR “patient education”[ti/ab] OR psychoeducation[ti/ab] OR “therapeutic education”[ti/ab] OR “consumer health information” OR “health knowledge”[ti/ab] OR “client education”[ti/ab]))
**Chronicity**	((chronic[ti/ab] OR “chronic disease”[ti/ab] OR long-term[ti/ab] OR "chronic disease"[MeSH] OR "chronic disease hospital"[MeSH])
**Disorders**	(respiratory[ti/ab] OR pulmonary[ti/ab] OR kidney[ti/ab] OR cerebrovascular[ti/ab] OR infection[ti/ab] OR cancer[ti/ab] OR metabolic[ti/ab] OR gastr*[ti/ab] OR cardiac[ti/ab] OR hypertension[ti/ab] OR asthma[ti/ab] OR COPD[ti/ab] OR neuro*[ti/ab] OR coronary[ti/ab] OR diabetes[ti/ab] OR hypertensive[ti/ab] OR urinary[ti/ab] OR urological[ti/ab] OR reproductive[ti/ab] OR cardiovascular[ti/ab] OR skin[ti/ab] OR dermatolog*[ti/ab] OR psychiatr*[ti/ab] OR mental[ti/ab] OR joint[ti/ab] OR hormon*[ti/ab] OR “heart disease”[ti/ab] OR disease*[ti/ab] OR endocrin*[ti/ab] OR neoplas*[ti/ab] OR communicable[ti/ab] OR non-communicable[ti/ab])
**Treatment outcomes**	(“Treatment outcome”[ti/ab] OR outcome[ti/ab] OR psychosocial[ti/ab] OR lab*[ti/ab] OR “physical outcome”[ti/ab] OR stress[ti/ab] OR depress*[ti/ab] OR “disease recurrence”[ti/ab] OR perception[ti/ab] OR “Disease progression”[ti/ab] OR “self-care”[ti/ab] OR complication*[ti/ab] OR hospitalization[ti/ab] OR self-efficacy[ti/ab] OR “self-management”[ti/ab] OR compliance[ti/ab] OR adherence[ti/ab] OR knowledge[ti/ab] OR attitude[ti/ab] OR behavior[ti/ab] OR “quality of life”[ti/ab])
**Study design**	(trial*[ti/ab] OR RCT[ti/ab] OR randomized-controlled[ti/ab] OR “cluster randomized controlled”[ti/ab] OR intervention[ti/ab] OR “clinical trial"[PT] OR “controlled clinical trial"[PT])

Supplementary Table S2: Characteristics of interventions included in the review

Author, year	Brief name	Why?	Disorder	What? (materials and procedures)	Who provided?	Delivery agent	How?	Format of delivery	When and How Much ?
Aiello 2015	Personalized diabetes risk assessments during ophthalmologic visits.	To determine effect on glycemic control.	Diabetes	Intervention patients received at each visit (1)-measurement of HbA1c level, blood pressure, and retinopathy severity; (2) graph showing risk for worsening retinopathy ; (3) risk assessment for renal disease and retinopathy; (4) graph plotting patient‚Äôs previous and current HbA1clevels; (6) education materials. Control group: standard care.	researchers, ophthalmologists.	Multidisciplinary team	Face to face.	Individual	April 2011- January 2013: every 12 weeks:
Al-Haj Mohd 2016	Educational program on medication adherence among adults with diabetes.	To improve medication adherence and help prevent chronic complications associated with diabetes.	Diabetes	Materials: slides adopted from the IDF education modules. Procedures: Intervention group received education session about diabetes and medication focusing on importance of adherence, followed by follow-up phone calls. Control group: standard care.	research investigators: assessments; diabetes nurses: education.	Multidisciplinary team	PowerPoint presentation based on slides adopted from IDF. Weekly telephone follow-up.	telephone	study: February - November 2015. 1 educational session: 30 min. Follow-up : 3 months.
Assuncao 2010	Promotion of physical activity and reduction of consumption of sweets and sodium among overweight and obese adults.	To help reducing weight and control risk factors of noncommunicable chronic diseases in this population.	obesity	Material: manual with photographs of portion sizes of prescribed foods. Procedures: Intervention group received: (a) dietetic prescription, (b) guidance on choosing and substituting foods, (c) encouragement to consume vegetables, fruit and low-fat foods; and to perform physical activity and f) promotion of follow up visits. Control group: usual care.	2 trained nutritionists.	allied health workers	individually, face to face, telephoning absentees 15 days after missed appointments.	mixed	June 2005 - December 2006
Blomfield 2014	Self-Help, Exercise and Diet using Internet Technology (SHED-IT) intervention for overweight and obese men.	To determine whether SHED-IT participants were able to implement key dietary messages.	obesity	Participants were randomized to intervention groups: 1- "SHED-IT Online" , using website CalorieKing and online food and exercise diary with feedbacks provided; or 2- "SHED-IT Resources" that received DVD, Weight Loss Handbooks, pedometer and lifestyle diary with no feedback; or 3-Wait-list control group.	Website User Guide developed by the research team.	research team	internet	internet	recruitment: July-August 2010. 3-month intervention; feedbacks weekly first month, fortnightly second month and once third month. 6-month follow up.
Cade 2009	Expert Patient Program (EPP), adapted for people with Type 2 diabetes.	To assess whether adapted EPP can be used to promote healthy eating to improve glycaemic control.	Diabetes	Following baseline assessment, intervention group learned how to cope with a long-term health problem, self-managing diabetes in terms of food intake, physical activity, blood glucose and blood pressure. Control group: standard care.	senior trainer worked alongside volunteer peer tutors.	peer leaders	face to face, in groups	group	Recruitment: October 2003 - October 2004. EPP: 2-h sessions, once a week, for 7 weeks; follow-up at 6 and 12 months after EPP.

Carter 2011	Online diabetes self-management intervention for urban African Americans with type 2 diabetes.	To enable medically underserved communities with diabetes to improve health outcomes and assume more responsibility for health.	Diabetes	Material: laptops.<br>Intervention group was provided with online modules: 1- self-management education; 2- health education on nutrition, physical activity etc; and 3- social networking enabling participants exchange of information.<br>Control group: standard care.	telehealth nurse	allied health workers	internet	internet	biweekly 30-minute video conferences
Chao 2014	Educational self-care intervention for older Taiwanese patients with Type 2 diabetes mellitus (DM).	To improve self care behaviors, medical outcomes and QoL in this population.	Diabetes	Material: booklet on DM<br>Procedures: Intervention group was provided general information about DM; specific dietary suggestions; self-care requirements related to DM (home care: exercise, medicine management, self-monitoring of blood glucose, foot care).<br>Control group: usual care.	nurses trained by researchers.	research team	in groups of 25-70 participants.	group	2005 - 2006: 1h session every week for 3-weeks; 6month follow-up.
Chao 2015	Integrated health management model for adults with diabetes.	To improve health of older adults with diabetes.	Diabetes	Intervention group was provided 1-health record establisment; 2-health evaluation; and 3-health management, including diet, exercise, skills training on health self-management, lectures on health and diabetes.<br>Control group received usual care.	specifically-trained community health-service center staff, researchers.	Multidisciplinary team	face to face, telephone. Individually or in groups.	Mixed	Recruitment: January -July 2009; program: at least once per month, over 18 months, and measurements were performed.
Chaveepojnkamjorn 2009	Self-help group (SHG) program for type 2 diabetic patients.	To determine effect of the program on QoL of these patients.	Diabetes	Intervention patients received SHG program, aiming to improve knowledge of DM, skills for dietary control and physical exercise, self-monitoring, motivation for experiences sharing and training skills for group leaders.<br>Control patients: usual care.	trained health staff.	allied health workers	face to face, in small groups	group	5 monthly sessions, 2h each; QoL assessment: at 12 and 24 weeks.
Chen 2016	Pharmaceutical care of elderly patients with poorly controlled type 2 diabetes mellitus (DM).	To improve glycemic control.	Diabetes	Following identification and resolution of drug-related problems, intervention participants were provided DM education including assessment of adherence to pillbox use and insulin injection technique.<br>Control group: standard care.	certified diabetes-educator pharmacist.	allied health workers	face to face or by phone.	Mixed	study: August 2011 - February 2012; 6 months of follow up.
Chow 2015	Home-based patient education on diabetes-related knowledge and medication adherence.	To improve understanding of DM and increase adherence to prescribed medications.	Diabetes	Material: information booklet on T2DM, food pyramid chart.<br>Procedure: Intervention patients received education on: 1- proper use of medications, side effects and importance of medication adherence ; 2- T2DM: pathophysiology, complications, meal planning, and lifestyle modifications.<br>Control group: usual care.	pharmacist trained by the senior pharmacist in the clinic.	allied health workers	face to face at home, or by phone.	Mixed	April 2013 - September 2013; each patient: two home visits, average time on each visit: 60 min.

Cox 2004	Hypoglycemia Anticipation, Awareness and Treatment Training (HAATT).	To help diabetic patients to manage better, reduce occurrence of mild hypoglycemia, and to avoid severe hypoglycemia (SH).	Hypoglycemia	All participants received material and education to self-monitore blood glucose data. In addition, HAATT participants received structured, psychoeducational program, learning about risk and consequences of SH, insulin kinetics, and how to recognize cues signaling hypoglycemia.	researchers.	research team	groups of 10 patients.	group	7-weekly sessions; for 6 months before and 1 to 6 and 13 to 18 months after, participants recorded occurence of hypoglycemia; 1-month pre- and post-intervention, participants completed daily diaries concerning diabetes management.
Farmanbar 2019	Intervention based on transtheoretical model (TTM) on physical activity (PA).	To determine effects on HbA1c in diabetic women	Diabetes	Material: pamphlets and training CD about exercise in diabetics. Procedure: Intervention patients received education based on TTM, following stages of decision making to behavior change.<br>Control group: usual care.	researchers.	research team	face to face.	Individual	4 stages of training during 4 weeks, each stage 50-60 min; during 3months after, text messages about importance of PA were sent to patients and their families.
Gagliardino 2013	Educational strategies to improve diabetes care (PRODIACOR).	To achieve a long-term improvement in clinical, metabolic and psychological outcomes.	Diabetes	Material: educational materials, program book. Procedures: 2x2 design addressed education of physicians and people with diabetes with strong emphasis on active patient participation and self-care.<br>Control group: no education.	trained educators.	allied health workers	groups of up to 10 patients.	group	four 90-120min weekly teaching units and a reinforcement session at 6 months.
Gillett 2010	Diabetes education and self management for ongoing and newly diagnosed (DESMOND).	To assess long term clinical effectiveness of this programme.	Diabetes	Material: food models, display boards. Procedures: Intervention patients received structured education and self management program; and were encouraged to decide on their own goals, mainly weight loss and smoking cessation.<br>Control group: usual care.	trained health care educators.	allied health workers	groups of 10 patients.	group	DESMOND started in 2004; program duration: 6 hours over 12 weeks. Assessments: baseline and at 12 months.
Glasgow 1997	Brief behavioral dietary intervention for patients with diabetes, delivered from medical office.	To improve important outcomes.	Diabetes	Intervention involved touchscreen computer-assisted assessment that provided immediate feedback on key barriers to dietary self-management, goal setting and problem-solving counselling. Follow-up phone calls.<br>Control group: usual care.	interventionists.	research team	computer, ,Äò,Äòtake home,Äô,Äô video, telephone follow-ups.	Mixed	one session. 12- month follow-up

Goudswaard 2004	Self-management education for patients with Type 2 diabetes taking maximal oral hypoglycaemic therapy.	To improve glycemic control and delay need for insulin therapy.	Diabetes	Intervention group received education on diabetes, compliance with actual medication, importance of blood glucose control, physical exercise and losing body weight; and nutritional advice.<br>Control group: usual care.	two skilled diabetes nurses	allied health workers	one-to-one sessions.	Individual	During 6-month period, 6 sessions at intervals of 3,Äì6 weeks, between 15 and 45 min each.
Howorka 2000	Empowering diabetes out-patients with structured education for Functional Insulin Treatment (FIT).	To improve patients,Äô perceived control over diabetes.	Diabetes	All participants received general diabetes education (phase 1). Intervention patients received in addition, structured training in FIT for selective use of insulin either for eating, fasting or correction of hyperglycemia using practical "insulin games" (phase 2).	researchers.	researchers	phase 1: individually.<br>phase 2: in groups.	Mixed	phase 1: 18 hours<br>phase 2: 35 hours
Huizinga 2010	Preventing glycaemic relapse in recently controlled type 2diabetes patients.	To improve management of diabetes.	Diabetes	All participants received routine follow-up. Intervention group was in addition helped to solve problems in self-care behaviors, including diet, self-monitoring of blood glucose, exercise, and medication adherence.	nurse practitioners and dietitians, certified in diabetes education, with clinical experience in providing diabetes care.	allied health workers	phone contacts.	telephone	enrollment: June 2002 - January 2005; contacts: monthly or quarterly, over 2 years.
Jarab 2012	Clinical pharmacy management of patients with Type 2 Diabetes.	To improve clinical outcomes.	Diabetes	Material: booklets.<br>Procedure: Intervention group received objective-directed education about T2DM and necessary lifestyle changes, followed by telephone follow-up calls.<br>Control group: usual care.	clinical pharmacist.	allied health workers	face-to-face education, telephone follow-up	Mixed	recruitment: January-April 2011.<br>baseline education followed by 8 weekly phone calls, about 20min each.
Kampan 2006	Counseling and implementation of clinical pathway on diabetic patients hospitalized with hypoglycemia.	To reduce length of hospital stay and decrease readmission rates of recurrent hypoglycemia.	Hypoglycemia	Following treatment of hypoglycemia, intervention group was provided education to manage diabetes at home, including recognition of hypo- and hyperglycemic symptoms and nutrition counseling.<br>Control group: usual care	nurse educator.	allied health workers	face to face to each patient and his or her family.	Individual	July - December 2005.
Ko 2007	Structured intensive diabetes education programme (SIDEP) in patients with diabetes mellitus (DM).	To maintain optimal glycaemic control, especially in insulin-treated patients.	Diabetes	During their stay in hospital, SIDEP patients were provided program focused on understanding of DM and its management. Family members invited to attend.<br>Control group: usual care.	diabetologist, certified diabetes educator, ophthalmologist, rehabilitation therapist, pharmacist, psychologist and rehabilitation medicine doctor, all professional health providers in field of DM.	Multidisciplinary	groups of 5- 10 patients	Group	6 h per day for 5 days during patients,Äô stay in hospital, total of 30 h; after discharge, they were followed up at 3 months intervals.

Korhonen 1983	Patient Education in the treatment of Insulin-dependent Diabetes.	To improve diabetes control.	Diabetes	Material: booklets.<br>Procedure: During hospitalization, intervention patients received education on diabetes and how to adjust insulin dose in special situations.<br>Control group: traditional education.	physicians, dietitian, teaching nurses who specialized in diabetes.	Multidisciplinary	both individually and in small groups	Mixed	over 5-day hospital stay: with physician: 1 h; teaching nurse: 8-12 h; dietitian: 2.5 h.
Kruger 1992	Foot care for adults with diabetes.	To improve foot care practices.	Diabetes	Material: video, foot check sheets.<br>Procedures: All participants received usual teaching on foot care. In addition, intervention patients were involved in hands-on teaching/learning foot care sessions.	technical assistants trained by nurses and podiatrists	Allied health workers	in groups.	Group	assessments: baseline and at 6-month.
Lee 2011	Diabetes mellitus (DM) self management programme.	To improve clinical outcomes, patient self efficacy and lifestyle behaviours.	Diabetes	Intervention patients were encouraged on healthy behaviors by promoting their problem solving skills related to personal lifestyle: healthy eating, being active, drug compliance, reducing risks, and healthy coping.<br>Control group: usual care with general advice on lifestyle and drug compliance.	social worker of the Community Rehabilitation Network (CRN), accredited as trainer for self management program.	Allied health workers	in small groups (12-15) for 2.5 h	Group	six weekly sessions, 2.5 h each.
Luan 2017	Diabetes education and self-management support of diabetes patients.	To improve self-management skills, blood glucose and lipid levels control and reduce medical costs.	Diabetes	Intervention group received education towards diabetes self-management support. Patients were helped setting goals and identify constructive solutions.<br>Control group: traditional diabetes education.	endocrinologists, diabetes-educating nurses, dieticians, psychological counsellors.	Multidisciplinary	face to face, individually.	Individual	monthly in-home follow-ups.
Lutes 2008	ASPIRE (Aspiring for Lifelong Health) program for weight loss treatments.	To examine efficacy of intervention targeting small but cumulative participant-chosen changes in diet and physical activity(PA).	Obesity	Intervention group was offered either ASPIRE focused on small, cumulative changes in PA and nutrition or standard educationally-based nutrition and PA program, both coupled with resistance and aerobic training.<br>Control group continued life as usual.	Aspire: lifestyle coaches ;<br>Standard: nutritionists.<br>Aerobic and resistance training: personal trainers.	Multidisciplinary	individually.	Individual	didactic behavioral counseling: total 5 h; meetings with nutritionist (Standard group) or lifestyle coach(ASPIRE): 20min, once a week over 16 weeks of study.<br>Each training session 40,Äì45 min, performed twice per week.<br>Total: 24 h of personal training supervision for each participant.

McGowan 2015	Self-Management programs for Type 2 Diabetes Mellitus (T2DM).	To assess two programs in bringing about improvements in subjects with T2DM.	Diabetes	T2DM patients were randomly allocated to CDSMP (Chronic Disease Self-Management Program) or DSMP (Diabetes Self-Management Program) learning on top of the same strategies all necessary skills required by persons living with T2DM.<br>Control group: usual care.	pairs of trained peer-leaders.	Peers	in groups.	Group	6-months program; once a week for 2.5 hours. Assessments: baseline, at 6 and 12 months post-program.
McKay 2001	Diabetes Network (D-Net) Physical Activity (PA) intervention.	Provide support for sedentary patients with type 2 diabetes to increase their PA levels.	Diabetes	Intervention patients were guided in goal-setting, received and could post messages to an on-line personal coach, and participated in peer group support areas.<br>Control group: Internet information-only.	personal coach (occupational therapist) with access to endocrinologist, registered dietitian, and exercise physiologist.	Allied health workers	Internet.	internet	Over 8-weeks.
McRobbie 2016	Task-based weight management group program: [Weight Action Programme (WAP)].	To provide participants with tools to lose weight and maintain a long-term healthy lifestyle.	Obesity	WAP aimed at encouraging and improving self-efficacy, monitoring of exercise levels; self-regulation through use of food diaries, self-monitoring of weight and goal-setting.<br>Control group received best usual care based on NHS ,ÄòChange4Life,Äô.	research health psychologists, group facilitators.	Allied health workers	face to face, in groups of 10-20.	Group	8 weekly sessions, followed by 10 monthly maintenance sessions.
Mohammadi 2018	Self-efficacy education in Iranian patients with type 2 diabetes.	To improve metabolic and glycemic profiles.	Diabetes	Material: diabetes booklet, knowledge and health questionnaires.<br>Procedure: Intervention patients were educated about diabetes and its complications, self-care and to self-monitor blood glucose. Control group: conventional dietary counseling only.	health professionals, diabetes specialist.	Multidisciplinary	in groups.	Group	12-week program:<br>8 sessions, 2h each.<br>24-week post-intervention follow-up; outcomes evaluated at baseline, week 12 and week 36.
Moriyama 2009	Self-management education program for people with type 2 diabetes.	To improve patients' health outcomes.	Diabetes	Following clinical examination, intervention patients received education about diet, exercise, smoking cessation, medication and stress management, prevention of diabetic complications.<br>Control group: usual care.	trained educators.	Allied health workers	face to face, individually.	Individual	12 months for each patient; monthly sessions, lasting~30 min.
Nejhaddadgar 2019	Self-management program for people with type 2 diabetes mellitus (T2DM) based on PRECEDE-PROCEDE model.	To enhance self-management behaviors.	Diabetes	Following assessments, intervention patients received program based on PRECEDE model (knowledge, attitudes, social and family support), to improve self-efficacy.<br>Control group: usual care.	facilitators ;<br>2-session facilitator training workshops were conducted among patients,Äô families and health workers as well.	Allied health workers	face to face.	Individual	8 weekly sessions; assessments: baseline and 6 months after intervention.
Nichter 2018	Smoking cessation for diabetes patients.	To prevent disease complications.	Diabetes	All participants were informed about danger of smoking. In addition, intervention group received diabetic specific tobacco counselling on diabetes complications exacerbated by smoking.	counsellor.	Allied health workers	face to face, individually.	Individual	3 sessions lasting 30minutes; 2 years follow-up.

Perri 2008	Treatment of Obesity in Underserved Rural Settings (TOURS).	To examine options for long-term weight management.	Obesity	Following completion of lifestyle treatment, participants randomized to: 1- Telephone Counseling; 2- Face-to-Face Counseling; 3- Education Control, all using the same handouts, addressing barriers to eating and exercise behaviors for sustaining lost weight.	same counselors who led initial lifestyle program.	Allied health workers	telephone or face to face.	Mixed	26 biweekly sessions, 15- to 20-minute each par telephone, 60minute each face to face.
Piatt 2010	Multifaceted diabetes care based on Chronic Care Model (CCM).	To assess long-term improvements in clinical, behavioral, and psychosocial outcomes.	Diabetes	1- CCM group: patient and provider education, as well as other CCM elements.<br>2- "Provider education only" group: Provider-based diabetes education at one problem-based learning session.<br>3- Control group: usual care.	certified diabetes educator (CDE).	Allied health workers	face to face, individually.	Individual	6 weekly sessions on provider-specified ‚Äúdiabetes days‚Äù, followed by monthly support groups held until 12th follow-up visit.
Rachmani 2005	Motivation and Teaching intervention for high-risk patients with Diabetes.	To attenuate course of microvascular and cardiovascular sequelae of diabetes.	Diabetes	Intervention patients were taught ways to achieve tight control of risk factors; individualized plan of lifestyle modification, fitness program.<br>Control group: usual care.	staff in diabetes clinic.	Multidisciplinary	face to face, individually.	Individual	two 2-h teaching sessions.<br>8-yr phase follow-up.
Ramadas 2018	Web-based dietary intervention for patients with type 2 diabetes (T2DM).	To improve health behavior changes.	Diabetes	Intervention patients were provided access to myDIDeA ; dietary lesson plans personalized according to patients‚Äô Dietary Stages of Change.<br>Control group: usual care.	study nutritionist.	Allied health workers	via website.	website	12 lesson plans made available to patients one after another over period of 6 months, with updates every fortnight.
Reid 2018	Ottawa Model for Smoking Cessation (OMSC) for T2DM patients.	To improve long-term abstinence among smokers with diabetes.	Diabetes	OMSC group received counseling, discount card to cover cost of smoking cessation medication, quit plan and follow-up.<br>Wait-List Control group: usual care for smoking cessation.	trained practice facilitators and diabetes educators.	Allied health workers	face to face, individually.	Individual	30 days, follow-up phone calls over 6-month period.
Riddell 2016	Lay peer led program for people with diabetes.	To improve control and management of type 2 diabetes (T2DM).	Diabetes	All participants attended diabetes self-management education<br>Intervention group in addition: assistance with the ‚Äúhow‚Äù of daily self-management and provision of social and emotional support.<br>Control group: usual care.	volunteer peer supporters trained by credentialed diabetes nurse educator experienced in group facilitation and communication.	Allied health workers	meetings in groups.	Group	12 monthly meetings.<br>Assessments: baseline, at 6- and 12-month.
Salinero-Fort 2011	PRECEDE (Predisposing, Reinforcing, Enabling, Causes in Educational Diagnosis, and Evaluation) model in patients with type 2 diabetes.	To decrease glycated hemoglobin HbA1c and BP levels and increase compliance in control criteria.	Diabetes	Intervention patients received Health Promotion Education (HPE) based on PRECEDE model: skills and instruments to reach behavior change.<br>Control group: conventional HPE.	Nurses prepared by researchers, trained in procedure to be used in 3 sessions.	Allied health workers	individually, face to face during visits.	Individual	2 baseline visits (month 1), 8 follow-up visits (every 3 months), 40 min per visit.

Schachinger 2005	Blood Glucose Awareness Training (BGAT): psycho-educational program for patients with type 1 diabetes mellitus.	To improve recognition and management of extreme blood glucose levels.	Diabetes	BGAT: training to recognize when BG is too high or low and anticipate when it is likely to rise or fall. Homework and readings required. Controls: physician-guided self-help group.	physician/psychologist team.	Multidisciplinary	in groups of 5,Äì12 subjects.	Group	8 weekly sessions of 2h each.
Schillinger 2009	Self-Management Support (SMS) for patients with poorly controlled diabetes.	To improve behavior and QoL of these patients.	Diabetes	1-Automated Telephone Self-Management (ATSM): education and patient activation linked to nurse care by phone; 2- Group Medical Visits (GMV): support, education, patient activation by group process. Both intervention groups promoted behavioral ‚Äúaction plans". Controls: usual care.	1-nurse (group ATSM); 2-physician and health educator(group GMV).	Multidisciplinary	ATSM: individually by telephone. GMV: sessions in groups of 6-10 participants.	Mixed	ATSM: weekly, over 9 months, each call 6-10 minutes. GMV: 90-min monthly over 9 months.
Seligman 2018	Diabetes Self-Management Support from food banks.	To support improvements in diabetes clinical outcomes.	Diabetes	Formal diabetes self-management classes and 1-on-1 check-ins with educators, food packages containing diabetes-appropriate foods and HbA1c testing. Waitlist control group received intervention after 6 months.	Educators: food bank staff trained in curriculum delivery by registered nurse and diabetes educator.	Multidisciplinary	face to face, individually.	Individual	6-month intervention; two 2-hour sessions (within the first 2months of enrollment) and monthly 1-hour drop-in.
Shea 2007	Informatics for Diabetes Education and Telemedicine: IDEATel project.	To assess telemedicine case management for diabetes.	Diabetes	Intervention patients: videoconferencing and remote monitoring of glucose and BP. Control group: usual care.	project case manager under supervision of diabetologists.	Allied health workers	internet.	internet	1 year.
Shibayama 2007	Lifestyle counseling for non-insulin-treated diabetic outpatients.	To improve patients,Äô health outcomes.	Diabetes	Intervention: 1-assessment of eating patterns, level of physical activity and self-care for diabetic complications; 2- goal setting; 3-follow-up including evaluation and feedback. Control group: usual care.	certified expert nurse.	Allied health workers	one-to-one.	Individual	counseling took 18,Äì76 min at monthly hospital visit during 1 year.
Soennichsen 2010	Austrian disease management program (DMP) for type 2 diabetes.	To evaluate effectiveness of this program on metabolic control and quality of care.	Diabetes	Patient education in 4 modules, structured interdisciplinary care, agreement on therapeutic goals in shared patient-physician decision-making process at 3- monthly intervals. Control group: usual care.	physicians that underwent 10-hour training course	Doctors	groups of 3 -12 patients.	Group	9 hours of patient-education. Assessments: baseline, and at 12 months.
SonalSekhar 2018	Patient-education (PE) in diabetic foot ulcer (DFU) patients.	To improve health related quality of life (HRQoL).	Diabetes	Education about foot care measures. Counseling on medication compliance, need for off-loading, wound dressing, properly fitting foot wear and annual podiatry reviews. Control group: usual care.	clinical pharmacists.	Allied health workers	individually, during visits; follow-up: monthly phone contacts.	Mixed	6 months.
Tourkmani 2018	Integrated care program for patients with type 2 diabetes.	To improve glycemic control and cardiovascular risk factors.	Diabetes	Material: booklet for home BG monitoring. Procedure: Intervention group was provided more frequent monitoring of outcomes, additional diabetic education and health counseling. Control group: standard care.	team: family physician, pharmacy specialist, dietician, diabetic educator, health educator, social worker.	Multidisciplinary team	individually, face to face.	Individual	between September 2013 and September 2014, at least weekly appointments in the first 3 months.

Trouilloud 2013	Therapeutic education among adults with type 2 diabetes.	To evaluate impact on perceived competence, self-management behaviors and glycaemic control.	Diabetes	Educational and problem-solving activities on 3 main components of diabetes management: diet, physical activity and medication.<br>Control group: waiting list during a 3-month period.	multi-professional team: diabetologist, dietician, nurses and physical activity counsellor.	Multidisciplinary	in groups of 5,Äì8 patients.	Group	3-day program:<br>8 sessions, 2,Äì3h each; follow-up: 3 months.<br>Assessments: baseline, at 3 months.
Wadden 2011	Lifestyle counseling (LC) in obesity treatment.	To help obese patients achieve long-term, clinically meaningful weight loss.	Obesity	All participants received pedometer, cal.-counting book, the same goals regarding diet and PA, and provided with LC or enhanced LC that included meal replacements or weight-loss medication.<br>Control group: usual education about weight management.	lifestyle coaches.	Allied health workers	individually, face to face or by phone.	Mixed	year 1: quarterly PCP visits combined with monthly 10-15min sessions with lifestyle coaches; year 2: every other month, counseling by phone.
Wagner 2001	Chronic care clinics for diabetes.	To evaluate process and outcome of care.	Diabetes	Chronic care clinics provided: assessments; visits with PC physician, nurse, and clinical pharmacist; group education/peer support meeting, covering self-management (SM).	PC physician, practice nurse, pharmacist.	Multidisciplinary	individual visits with PC physician, one-on-one counseling with nurse, group session for SM.	Mixed	groups of 6 ,Äì10 invited to attend at intervals of 3,Äì 6 months.
Whitehead 2017	Education intervention among adults with uncontrolled type 2 diabetes.	To assess nurse,Äêled educational intervention alone and nurse,Äêled intervention using education and acceptance and commitment therapy (ACT).	Diabetes	Education about diabetes, risk factors, nutrition,self,Äêmanagement through diet, exercise, medication, and stress management. ACT addressed mindfulness and acceptance training in relation to difficult thoughts and feelings.<br>Controls: usual care.	education: nurses trained by study investigators.<br>ACT: mental health nurse with expertise in ACT supervised by clinical psychologist.	Multidisciplinary	in group.	Group	1 day workshop.
Williams 2012	Telephone-Linked Care (TLC) Diabetes system.	To improve type 2 diabetes management.	Diabetes	Material: Handbook, glucose meter, test strips, Bluetooth.<br>Intervention participants were asked to call TLC;<br>BG, nutrition, physical activity and medication-taking were targeted.<br>Control group: usual care.	health professionals	Multidisciplinary	telephone	Telephone	weekly calls over 6 months, calls lasting 5-20 min depending on call content.

Supplementary Table S3: Differences in content of interventions according to modes of delivery using Kruskal-Wallis test

	Disease management	Lifestyle changes	Coping skills	Disease processes	Interpersonal skills
Kruskal-Wallis H	4.255	5.852	4.906	6.273	0.391
P-value	0.235	0.119	0.179	0.099	0.942
